# Application of a nanotechnology antimicrobial spray to prevent lower urinary tract infection: a multicenter urology trial

**DOI:** 10.1186/1479-5876-10-S1-S14

**Published:** 2012-09-19

**Authors:** Wei He, Dongmin Wang, Zhangqun Ye, Weihong Qian, Yan Tao, Xiaofeng Shi, Ling Liu, Jin Chen, Ling Qiu, Peng Wan, Xiaojun Jia, Xia Li, Caixia Gao, Xuexia Ma, Biyan Wen, Nianzhen Chen, Ping Li, Zhengzheng Ren, Li Lan, Siyi Li, Yi Zuo, Hua Zhang, Liming Ma, Yueping Zhang, Zhicong Li, Weiping Su, Qing Yang, Qingli Chen, Xuejing Wang, Zhenni Ye, JP Chen, Wings TY Loo, Louis WC Chow, Adrian YS Yip, Elizabeth LY Ng, Mary NB Cheung, Zhiping Wang

**Affiliations:** 1 Department of Urology, Tongji Hospital Affiliated to Tongji Medical College, Huazhong University of Science & Technology, Hubei PRC; 2 Institute of Urology, the Second Hospital of Lanzhou University, Lanzhou, PRC; 3 Wuhan General Hospital of Guangzhou Military Region, Guangzhou, PRC; 4 West China Hospital, Sichuan University, Sichuan, PRC; 5 Daping Hospital, Third Military Medical University, Chongqing, PRC; 6 The First Affiliated Hospital of Guangzhou Medical University, Guangzhou, PRC; 7 The Second Military Medical University, Changhai Hospital, Shanghai, PRC; 8 Peking University People's Hospital, Beijing, PRC; 9 The Third Affiliated Hospital, Sun Yat-sen University, Guangdong, PRC; 10 The Second Hospital of Xi'an Jiaotong University, Xi’an, PRC; 11 The Second Affiliated Hospital, Sun Yat-sen University, Guangzhou, PRC; 12 Xiangya Hospital of Central-south University, Changsha, PRC; 13 Nanjing Drum Tower Hospital, The Affiliated Hospital of Nanjing University Medical School, Nanjing, PRC; 14 Huai'an First Hospital Affiliated to Nanjing Medical University, Jiangsu, PRC; 15 The First Affiliated Hospital, Sun Yat-sen University, Guangzhou, PRC; 16 The Second Affiliated Hospital of Guangzhou University of Traditional Chinese Medicine, Guangzhou, PRC; 17 The First Affiliated Hospital of Southern Medical University (Nanfang Hospital), Guangzhou, PRC; 18 Affiliated Hospital of Nantong University, Jiangsu, PRC; 19 Foshan Hospital of Traditional Chinese Medicine, Guangdong, PRC; 20 Guangzhou First Municipal People's Hospital, Guangzhou, PRC; 21 General Hospital of Guangzhou Military Command of PLA, Guangzhou, PRC; 22 The First Affiliated Hospital of Nanjing Medical University, Jiangsu, PRC; 23 The Second Affiliated Hospital of Kunming Medical College, Yunnan, PRC; 24 School of Chinese Medicine, The University of Hong Kong, Hong Kong SAR; 25 UNIMED Medical Institute, Hong Kong SAR

## Abstract

**Background:**

Catheter-associated urinary tract infection (CAUTI) is a common nosocomial device-associated infection. It is now recognized that the high infection rates were caused by the formation of biofilm on the surface of the catheters that decreases the susceptibility to antibiotics and results in anti-microbial resistance.

In this study, we performed an *in vitro* test to explore the mechanism of biofilm formation and subsequently conducted a multi-center clinical trial to investigate the efficacy of CAUTI prevention with the application of JUC, a nanotechnology antimicrobial spray.

**Methods:**

Siliconized latex urinary catheters were cut into fragments and sterilized by autoclaving. The sterilized sample fragments were randomly divided into the therapy and control group, whereby they were sprayed with JUC and distilled water respectively and dried before use.

The experimental standard strains of *Escherichia coli* (*E. coli*) were isolated from the urine samples of patients. At 16 hours and 7 days of incubation, the samples were extracted for confocal laser scanning microscopy.

A total of 1,150 patients were accrued in the clinical study. Patients were randomized according to the order of surgical treatment. The odd array of patients was assigned as the therapy group (JUC), and the even array of patients was assigned as the control group (normal saline).

**Results:**

After 16 hours of culture, bacterial biofilm formed on the surface of sample fragments from the control group. In the therapy group, no bacterial biofilm formation was observed on the sample fragments. No significant increase in bacterial colony count was observed in the therapy group after 7 days of incubation.

On the 7th day of catheterization, urine samples were collected for bacterial culture before extubation. Significant difference was observed in the incidence of bacteriuria between the therapy group and control group (4.52% *vs.* 13.04%, *p* < 0.001).

**Conclusions:**

In this study, the effectiveness of JUC in preventing CAUTI in a hospital setting was demonstrated in both* in vitro* and clinical studies.

## Background

Catheter-associated urinary tract infection (CAUTI) is a common nosocomial device-associated infection. Urinary tract infection (UTI) accounts for up to 40% of nosocomial infections and is one of the main types of healthcare-associated infections (HAI). About 80% of UTIs are catheter-associated [1,2]. In the United States, approximately 95% of UTIs were associated with the indwelling catheters [3], and interestingly, 15-25% of patients in short-term hospital care need to be inserted with indwelling urinary catheters [4]. Every year, there are more than 5 million patients necessitating catheterization therapy [5] and approximately 1 million patients suffering from CAUTI [6]. The findings of a European study indicated that 5.4% of patients aged 65 or above required the use of an indwelling urinary catheter [7].

CAUTI is a highly common infection and comes with considerable risk. The duration of hospitalization owing to CAUTI increased from 2.4 to 5.4 days in the United States [8]. On average, the costs of diagnosing and treating CAUTI is US$ 589, excluding extension of hospital costs [9]. Taking into account the expenses of hospitalization, the average cost increases from US$ 2,836 to 3,803 [10,11]. The Centers for Disease Control and Prevention (CDC) pointed out that UTI leads to deaths of over 13,000 patients every year in the United States [12], indicating a growing medical problem.

It is now recognized that the high infection rates were caused by the formation of biofilm on the surface of the catheters that decreases the susceptibility to antibiotics and results in anti-microbial resistance [8,13,14]. The formation of biofilm as a result of extracellular polysaccharide matrix secretions from microorganisms has been demonstrated in clinical studies. Bacterial biofilm is a special honeycomb-shaped structure that forms a very complex ecosystem; magnification of biofilm will reveal microcolonies under the microscope [15-18]. Organisms with biofilm can withstand shear force, pH changes, and antimicrobial agents, and prevent macrophage phagocytosis [13,19]. The proximity of cells allows more frequent genetic information exchange than other free cells [20]. Therefore, antimicrobial resistance genes and strains can be spread easily. With respect to catheters, the formation of biofilm will protect the pathogenic bacteria residing at the urinary tract from antimicrobial medicine and host immune response [15]. It will then facilitate the growth of bacteria which further complicates the problem of CAUTI [13].

Recent research focused on the development of preventive methods for biofilm formation and changes, including furanone, furacilinum, silver-coated catheters, in addition to other techniques. [21-24]. Johnson *et al*. [21,2] discovered that catheters containing silver hydrogel and nitrofurazone coating have excellent effects of inhibiting biofilm formation, but no inhibitory effects for *Pseudomonas aeruginosa*. According to the study conducted by CDC, the results of a comparison of patients inserted with silver-coated catheters and standard catheters for one week revealed no difference in bacteriuria prevention [25]. Silver-bearing catheters can decrease the effect of bacteriuria in a week after indwelling. Burton *et al.*[8] discovered the new oPDM-plus-PS (N, N'-(1,2-phenylene) dimaleimide [oPDM]-plus-protamine sulfate [PS]) coating can inhibit *Pseudomonas aeruginosa* and *Staphylococcus epidermidis* adhering to the catheters, but now these coated catheters can only provide short-term CAUTI prevention upon urinary catheter insertion [13]. Recently, Stickler *et al.*[26] revealed that bacteria on the biofilm of catheters produced quorum-sensing signal that can control the genetic expression of forming biofilm. If the signal is blocked, the formation of biofilm can be impeded. For example, the mutant *Pseudomonas aeruginosa* in the absence of quorum-sensing signal was unable to produce a three-dimensional biofilm [27]. An important finding was established regarding iron and the formation of biofilm. Clinical investigations have detected that elements such as iron are necessary nutrients for biofilm formation. The production of catheters without iron is a new development, but it has not been tested in clinical trials [13]. The use of probiotics can also be considered. Trautner *et al.*[28,29] observed that the rates of pathogenic bacterial infection and CAUTI were reduced if the catheters were inoculated with the non-pathogenic *Escherichia coli* (*E. coli*). Although these methods are considerable, there is no conclusive evidence and the cost-effectiveness remains unclear.

The traditional use of JUC applied to the wounds of post-surgery patients has proven to be effective in the hospital and out-patient setting: application of JUC did not result in drug resistance, nor stimulate serious adverse reactions and reduced the average wound healing time of patients [30]. JUC is composed by nano-manufacture technology, with nano-cations on the nano-scale molecular structure produced and then prepared in water-soluble spray [31]. JUC achieves antibacterial action on skin and wound surface by physical mechanisms and can therefore be regarded as a physical antimicrobial agent [31]. Upon application, JUC prevents bacterial growth by forming an invisible, positively charged protective film on the sprayed surface, isolating and eradicating negatively charged pathogenic micro-organisms including bacteria, fungi and viruses [31,32].

There is no effective way to prohibit biofilm formation clinically; therefore, there is still an unmet need for the establishment of a new clinical application. In this study, we performed an *in vitro* test to explore the mechanism of biofilm formation and subsequently conducted a multicenter clinical trial to investigate the efficacy of CAUTI prevention with the application of JUC, a nanotechnology antimicrobial spray.

## Methods

### *In vitro* testing

#### Bacteria

The experimental standard strains of *E. coli* were isolated from the urine samples of UTI patients at the Second Hospital of Lanzhou University. Bacteria were cultured in Luria-Bertaini broth [33]. The bacterial suspension was prepared and the bacterial concentration was adjusted to 7.4 × 10^9^CFU/ml.

#### Preparation of sample fragment

Siliconized latex urinary catheters were cut into sample fragments and sterilized by autoclaving. The sterilized sample fragments were randomly divided between the therapy and control group, with 8 pieces of fragments in each group. The sample fragments were respectively sprayed with JUC and distilled water, and dried before use. The *E. coli* suspension was injected into 24 well plates in which the sample fragments were placed. The plates were then incubated at 37 °C and washed with PBS solution every 48 hours [34]. At 16 hours and 7 days of incubation, the samples were extracted for confocal laser scanning microscopy.

#### Confocal laser scanning microscopy

The cultured samples were soaked in 1 ml PBS solution. After 50 μg/ml propidium iodide was added, the samples were left for dyeing in a dark area at 4°C for 15 minutes or at room temperature for 30 minutes. The samples were then placed upside down on a glass slide for observing the biofilm formation using laser scanning microscopy [35,36].

#### Clinical trial

The clinical study commenced in March 2010 and was completed in December 2011. Patients undergoing urological surgery in need of indwelling urethral catheter and more than 7 days of hospitalization were recruited. A total of 1,150 patients (869 male and 281 female), aged from 2 to 82 years, were accrued. Twenty-three hospitals participated in this clinical trial, and every hospital accrued 25 patients each to the control group and therapy group. All patients were operated due to urological diseases, including but not limited to urinary tract stones, tumors, prostatic hyperplasia, ureteral stenosis and hydronephrosis. Indwelling urethral catheter was necessary for patients requiring over 7 days of hospital stay. The midstream urine bacterial culture [15-17] was negative at the time of inclusion in the study. Exclusion criteria of the study included patients with a long-term use of balloon catheter, intermittent self-catheterization, previous treatment of percutaneous paracentetic suprapubic cyctostomy and UTI patients. Patients were randomized according to the order of surgical treatment. The odd array of patients (575 cases) was assigned as the therapy group (the JUC), and the even array of patients (575 cases) was assigned as the control group (normal saline). Patients who were eligible for the trial were explained the nature and purpose of the trial by the investigator, and informed consent was obtained for inclusion in the trial. The Ethics Committee of Tongji Hospital approved the clinical study (Approval Number: 2010006D).

### Study design

#### Therapy group

Prior to the insertion of the catheter into the ureter of the patient at the time of surgery, JUC was sprayed on the surface of the catheter to allow formation of a physical anti-microbial membrane. After surgery, in addition to traditional nursing care, JUC was sprayed onto the skin and mucous membrane around the urethral orifice, the catheter and the drainage tube attachment point. This was done twice a day with 1 ml per spray (approximately 10 sprays) until the catheter was removed on the 7th day.

#### Control group

The catheter was inserted during surgery. After surgery, conventional nursing care with normal saline was performed until the catheter was removed on the 7th day.

During the study, and according to routine clinical practice, antibiotics were prescribed to patients after surgery. The types, dosage and route of antibiotics prescribed to the patients were carefully and strictly recorded according to the class of antibiotics per institutional guidelines (Table 1).

**Table 1:** Classification of antibiotics used in the clinical trial

Class	Antimicrobial agents	Types	Usage Frequency	Rate of Usage
1	piperacillin, nafcillin, mezlocillin, azlocillin, ticarcillin, mezlocillin, amoxicillin, cefazolin, ceftazidime, cefathiamidine, cefprozil, cefixime, cefotiam, ceftriaxone, cefaclor, Cefonicid sodium, cefamandole sulfate, azithromycin, levofloxacin, ciprofloxacin,Lomefloxacin, enoxacin, gatifloxacin, amikacin, Amikacin’ Thiamphenicol, clindamycin	27	521	40.08%

2	ampicillin / sulbactam sodium, timentin / clavulanate, mezlocillin / sulbactam, amoxicillin / clavulanic acid, amoxicillin / sulbactam sodium, piperacillin / sulbactam sodium, cefuroxime sodium, cefmenoxime, cefotaxime sodium,cefpiramide, cefminox, cefodizime, cefpodoxime proxetil, cefetamet pivoxil, cefdinir, aztreonam, latamoxef sodium, cefoxitin sodium, sparfloxacin, moxifloxacin, fleroxacin, antofloxacin hydrochloride, tosufloxacin, etimicin, sisomicin, fusidate sodium, ornidazole	27	572	44%

3	Ceftizoxime, ceftazidime, ceftazidime, cefoperazone, cefoperazone / sulbactam sodium, ceftriaxone / sulbactam sodium, ceftriaxone / sulbactam sodium, cefoperazone / tazobatan, cefepime, cefoselis, imipenem / cilastatin, meropenem, Norvancomycin	13	207	15.92%

Class one, class two and class three antibiotics were cumulatively given 521 (40.08%), 572 (44%) and 207 (15.92%) treatment times respectively. There were no restrictions of use for class one antibiotics: they were proven to be safe and effective for long-term clinical application with minimal effects on antimicrobial resistance. The drugs belonging to class one are considered relatively inexpensive antimicrobial agents. Class two antibiotics demonstrated properties of restricted use, with concerned safety, efficacy, and antimicrobial resistance in humans. In comparison, class two drugs were relatively more expensive than the drugs of class one, non-restricted use antibiotics. Class three antibiotics are newly approved, antimicrobial agents with limited safety and efficacy information. There were reported adverse reactions with the use of class three antibiotics. Owing to the concerned safety of class three drugs, they are not recommended for use. Special attention should be made for clinical use to avoid bacterial resistance to antimicrobial agents. Amongst the three classes of antibiotics, class three are relatively more expensive in nature.

The clinical practice on the use of antibiotics differed between all hospitals. A total of 150 patients required the combination use of antibiotics, which were prescribed for 0 to 7 days according to the condition of the patient. The percentage use of class one antibiotics in Guangzhou First Municipal People's Hospital and The First Affiliated Hospital of the Sun Yat-sen University was 97.92% and 94.12% respectively, but the use of class two antibiotics was 97.40% in Daping Hospital of the Third Military Medical University. No significant difference was observed in the use of antibiotics between therapy and control groups in each hospital. For example, in the Second Hospital of Xi'an Jiaotong University, class one antibiotics were given to 10 cases in both therapy and control groups, class two antibiotics were given to 12 and 18 cases in treatment and control groups respectively, and class three antibiotics were given to 8 and 6 cases in treatment and control groups respectively. In the General Hospital of Guangzhou Military Command of PLA, class one antibiotics were given to 10 cases in both the therapy and control groups, class two antibiotics were given to 13 and 16 cases in the treatment and control groups respectively, and class three antibiotics were given to 4 and 3 cases in treatment and control groups respectively. Despite the difference in the clinical practice on the use of antibiotics between hospitals, the results were statistically meaningful.

After surgery, the body temperature and UTI symptoms were recorded every day. After 7 days of catheterization, urine samples were collected under aseptic condition for bacterial culture before extubation [37].

### Outcome assessment

The collected urine samples with colony count ≥ 10^3^ CFU/ml was considered as CAUTI, based on the quantitative urine culture. [9,38,39].

### Statistical analysis

Parameters were compared using SPSS version 14.0. The T-test was used to compare the incidence of CAUTI between groups, where P < 0.05 was considered as statistically significant.

## Results

### *In vitro* test results

**Figure 1:**
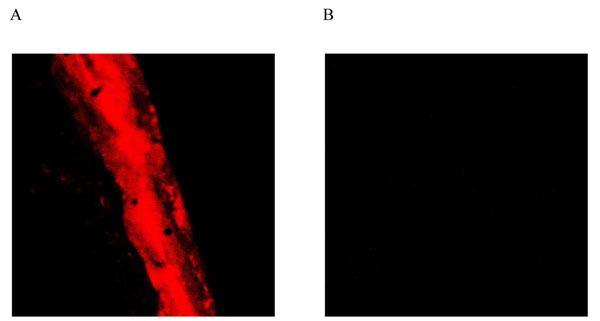
**Control and JUC group at 16 hours** A: Control group at 16 hours (CSLM 200X). B: JUC group at 16 hours (CSLM 200X)

After 16 hours of culture, bacterial biofilm formed on the surface of sample fragment in the control group. The bacterial biofilm was dyed red by propidium iodide fluorescent dye (Figure 1A). In the therapy group, no bacterial biofilm formation was observed on the sample fragments. Only small red dots representing a very small number of free bacteria were observed under microscope (Figure 1B).

**Figure 2:**
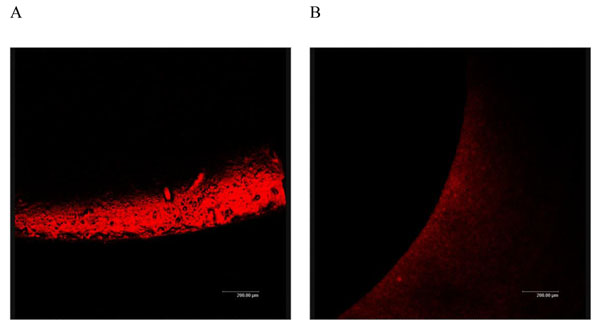
**Control and JUC group at 7 days** A: Control group at 7 days (CSLM 200X). B: JUC group at 7 days (CSLM 200X).

After 7 days of culture, in the control group with distilled water, the surface of the sample fragments formed a thick, uniform color, dense and darkly stained layer of bacterial biofilm. Due to bacterial overgrowth, the biofilm was cross-linked to form clumps of bacteria and the surface of the sample fragments was rough and uneven (Figure 2A). The surface of sample fragments in the therapy group formed only a small amount of thin membranous structure with smooth surface and light color. No other abnormality was observed (Figure 2B).

### Clinical trial results

Significant difference was not observed in demographics including age, gender, etiology, and geographical distribution between the two groups. On the 7th day of catheterization, urine samples were collected for bacterial culture before extubation. In the therapy group, positive bacterial culture was detected in 26 (4.52%) cases, of which 24 cases were *E. coli*, 1 case was *Enterococcus faecalis* and 1 case was *smooth Candida*. In the control group, bacteriuria was detected in 75 (13.04%) cases, of which 69 cases were *E. coli*, 2 cases were *Enterococcus faecalis*, 2 cases were *Enterococcus cloacae*, 1 case was *Candida albicans* and 1 case was *Pseudomonas aeruginosa*. Detailed results were shown in Table 2. Among all 101 cases of infections, 93 (92.08%) cases were *E. coli* infections, 3 (2.97%) cases were *Enterococcus faecalis*, and 2 (1.98%) cases were *Enterococcus cloacae*. Significant difference was observed in the incidence of bacteriuria between the control group and control group (4.52% *vs.* 13.04%, *p* < 0.001).

**Table 2:** Comparison of post-operative urinary bacterial culture between the control and therapy group

Groups	Number of Case	Before Surgery	Day 7 after surgery	Types of bacteria					
				
				*Escherichia coli*	*Enterococcus faecalis*	*Enterococcus cloacae*	*Candida albicans*	*Candida glabrata*	*Pseudomonas aeruginosa*
Therapy	575	0	26 (4.52%)*	24	1	0	0	0	1

Control	575	0	75 (13.04%)	69	2	2	1	0	1

* P<0.001, statistically significant

## Discussion

### Types of bacteria

UTI is a major nosocomial infection. CAUTI is one of the most common types of bacterial infections [1,2]; ample intestinal bacteria cultivates around the urethra [3,38]. A majority of the short-term CAUTIs were caused by a single strain of bacteria, such as *E. coli*, *Proteus mirabilis and Klebsiella pneumoniae*, whereas long-term CAUTI was caused by multiple microorganisms [3,40,41]. Urethra pathogenic *E. coli* is the most common cause of CAUTI which constitutes 50% of hospital-acquired UTIs [3,42]. In our study, similar results were observed. *E. coli* infection was dominated by 92.08%, while other bacteria such as *Enterococcus faecalis* and *Enterococcus cloacae* constituted a minute proportion of UTIs.

### Prevention of catheter-associated infection

Twenty years ago, the U.S. CDC clearly emphasized that hand hygiene, sterile catheterization and closed drainage systems were the necessary elements in preventing CAUTI [43,44]. Recently, the Healthcare-Associated Infections Allied Task Force proposed several frameworks, including infection surveillance, enhancement of education and training in the prevention of CAUTI, the use of appropriate technology for catheter insertion, replacement of indwelling urinary catheter by condoms and intermittent catheterization, immediate removal of the catheter, and other frameworks to prevent the occurrence of CAUTI [43,45,46].

The World Health Organization claimed systemic prophylactic antibiotic, irrigation of bladder, instilling normal saline or antibiotics, sterile drainage bag and other measures are ineffective in preventing the occurrence of CAUTI [1]. The use of anti-microbial drugs, anti-microbial drainage bag and irrigation of bladder can only temporarily reduce the chance of bacteriuria [13]. Furthermore, some studies have shown that the use of soap, skin cleansing foam, povidone iodine or saline in perineal care do not affect the incidence of CAUTI [47]. As for materials of the catheter, the single biological surface coated with silicon, polyurethane, synthetic biomaterials, or hydrogel material, were not proven effective in the prevention of bacterial colonization [5,16].

The formation of biofilm is regarded as one of the major causes of anti-microbial resistance and refractory CAUTI [13]. Therefore, the prevention of biofilm formation has been the research focus toward reducing the incidence of CAUTI. In this study, we investigated the use of new nanotechnology anti-microbial spray JUC, composed of organic silicon quaternary ammonium salt. JUC forms a positively charged film which isolates and kills negatively charged pathogenic micro-organisms including bacteria, fungi and viruses. The physical attraction between the film and the micro-organism would not lead to drug resistance [30].

In the laboratory, the formation of biofilm can be initiated by a small amount of bacteria. The bacteria clump together and form bacterial colonies. It then starts to form a biofilm. When the biofilm matures, it begins to shrink and collapse [48,49]. In the *in vitro* study, the initial stage of biofilm formation was observed at 16 hours of bacterial culture in the control group. After 7 days of incubation, aggregation of bacterial clumps was observed and the surface of sample fragments was unevenly rough, which illustrated the formation of mature biofilm. However, only few free bacteria, represented by red dots under microscope, were also observed in the therapy group. A thin membranous structure was observed which was at a stage between the bacterial colony formation and the initial formation of biofilm. The *in vitro* study clearly demonstrated that the physical anti-microbial film formed by JUC could prevent biofilm formation for 7 days upon application, which was validated in the clinical study. A significantly lower incidence of CAUTI was observed clinically in the therapy group (4.52%) than in the control group (13.04%), which further confirmed the effectiveness of JUC in the prevention of CAUTI.

### Comparison between clinical trials

The pre-operative use of anti-microbial drugs as an effective way for the prevention of bacterial infection is widely accepted [50]. In many clinical trials, prophylactic antibiotics were commonly prescribed for the prevention of infection in catheterized patients [5]. The UTI rate did not exclude the factor of antibiotics use. In Tambyah’s study [6], the mean antibiotics use was 1.6 ± 1.7 per catheter-day, and the incidence of CAUTI was 14.9% in the urology department. In the surgical unit, 1,162 patients were catheterized patients after surgery. The onset of CAUTI was 6.4 ± 6.1 catheterized days, and the incidence was 11.9% [6]. In Darouiche’s study [51], 124 patients were catheterized in place for 14 days with regular silicone bladder catheters or silicone bladder catheters impregnated with minocycline and rifampin after radical prostatectomy. All patients were given a single parental dose of 1g cefazolin as prophylactic antibiotic before anesthesia. The UTI rates measured 7 days after surgery were 15.2% and 39.7% in patients with regular catheters and medicated catheters respectively. The incidence was much higher than the therapy or control group patients of our study.

Compared to the study of 1,497 patients with an overall incidence of CAUTI of 14.9% by Tambyah *et al.*[6], the incidence of CAUTI was higher than the therapy group (4.52%) and slightly higher than the control group (13.04%) of our study. In Tambyah’s study, the patients were catheterized with nitrofurazone-impregnated silicone catheters, silver-polyurethane hydrogel catheters or control catheters and obvious differences were not observed in the incidence of UTI between medicated catheters and control catheters. Despite the different practices of the use of antibiotics between Tambyah’s study and our study, it seems the duration and types of post-operative antibiotics were not associated with the incidence of CAUTI. However, a significantly reduced incidence of CAUTI was observed in the therapy group of our study, indicating that the use of JUC, which was effective in preventing the biofilm formation, could be vital to lowering the incidence of CAUTI.

## Conclusions

In the clinical trial, only 4.52% of the patients from the therapy group were diagnosed with CAUTI, compared to 13.04% from the control group. *In vitro* testing also showed no obvious biofilm formation in the therapy group sprayed with JUC after 7 days of bacterial incubation. Biofilm began forming after 16 hours of incubation in the control group. The results from the clinical trial and *in vitro* test demonstrated the effectiveness of JUC in the prevention of CAUTI and formation of biofilm.

## Competing interests

The authors state they have no competing interests to declare.

## Authors' contributions

QC, NC, JC, JPC, CG, WH, XJ, LL, ZL, SL, XL, PL, LL, XM, LM, WQ, LQ, ZR, XS, WS, YT, PW, XW, DW, ZW, BW, QY, ZY, ZY, YZ, HZ, YZ equally conducted clinical test planning and performance. LWCC, WTYL, MNBC, AYSY and ELYN participated in the writing of the manuscript.
